# Inactivation of Yes-Associated Protein Mediates Trophoblast Dysfunction: A New Mechanism of Pregnancy Loss Associated with Anti-Phospholipid Antibodies?

**DOI:** 10.3390/biomedicines10123296

**Published:** 2022-12-19

**Authors:** Zengshu Huang, Zhijing Tang, Haiyun Guan, Wingting Leung, Lu Wang, Hexia Xia, Wei Zhang

**Affiliations:** 1 Obstetrics and Gynecology Hospital, Fudan University, Shanghai 200011, China; 2 Shanghai Key Laboratory of Female Reproductive Endocrine Related Diseases, Shanghai 200011, China

**Keywords:** antiphospholipid antibody, trophoblast, Yes-associated protein, cell biological function, progesterone

## Abstract

Pregnancy morbidity induced by anti-phospholipid antibodies (aPL+/PM+) is mainly thought to arise from placental abnormalities. We attempted to investigate the effect of aPL on the activity of Yes-associated protein (YAP) in the trophoblast and how YAP regulated human trophoblasts function. Thus, HTR-8 cells were treated with IgG purified from aPL+/PM+ women or normal controls. We found that aPL+/PM+ IgG impacted YAP activity via abrogating YAP expression. Further investigation of the anti-β2GPI-IgG/β2GPI complex showed an inhibition of nuclear YAP level and translocation in a dose-dependent manner, which might be rescued by progesterone in HTR-8 cells. YAP overexpression or knockdown HTR-8 cells were established for the evaluation of cell function and related gene expression in vitro. Loss of YAP arrested cell cycles in the G2/M phase, accelerated cell apoptosis by increasing the ratio of Bax/Bcl2, and disrupted MMP2/9-mediated cell migration and angiogenesis tube formation by VEGF. These findings support a new mechanism of PM associated with aPL through which YAP inactivation induced by aPL perturbs the trophoblast cell cycle, apoptosis, migration, and angiogenesis, finally developing into pregnancy failure.

## 1. Introduction

Antiphospholipid antibodies (aPL) are a heterogeneous population of autoantibodies that recognize phospholipid-binding proteins interacting with anionic phospholipids, such as β2-glycoprotein I (β2GPI) [1]. They are currently categorized into three main subtypes: anti-beta2 glycoprotein I (aβ2GPI), anti-cardiolipin (aCL), and lupus anti-coagulant (LAC) [2]. Persistent aPL positivity in serum has an intense relationship with anti-phospholipid syndrome (APS), a systemic autoimmune disease clinically characterized by thrombotic events and/or pregnancy morbidity [3]. Multiple case–control studies have shown that up to 50% of patients with pre-eclampsia (PE) or fetal intrauterine growth restriction (IUFGR) can be detected for aPL positivity in serum, compared with 7% or less in healthy pregnant women [4]. Another two meta-analyses concluded that aPL is a risk factor for consecutive early and late pregnancy losses [5,6]. It is considered that subsets and combinations of aPL contain certain value in predicting the risk of adverse pregnancy outcomes in aPL carriers [7,8].

Although human placental injury provoked by aPL was initially reported as thrombosis and infarction at the maternal–fetal interface, subsequent large-scale histological studies revealed that the fingerprint of placental change related to aPL involved trophoblast dysfunction and death, impaired spiral artery remodeling, and placental inflammation [9]. With research progress on the pathogenic mechanisms of APS subtypes, more clues point to discrepancies in pathogenicity between aPL+ with vascular thrombosis (VT) and aPL+ with pregnancy morbidity (PM) [10,11]. There is a significant difference in the N-glycan profiles of purified aβ2GPI IgG associated with thrombotic and obstetric APS: higher galactosylation in VT but lower galactosylation in PM [12]. In addition to thrombosis, aPL-induced pregnancy complications arise from failures in embryo development initiated by the attack of aPL on the trophoblast. β2GPI-dependent antibodies exert a role on placenta biology, appearing to be the central pathogenesis in APS [13]. They recognize the trophoblast cell surface protein β2GPI and then start the process of damage generation [14]: attenuating the proliferation and migration of the trophoblast via ApoER2 [15], eliciting secretion of pro-inflammatory cytokines such as IL-8 [16,17], weakening the invasion through MAPK [18], and inhibiting the secretion of β human chorionic gonadotropin hormone (β-hCG) and proangiogenic factors through TLRs [10,19,20]. The occurrence of aPL-positive adverse pregnancy outcomes might be a result of joint action of aPL on the maternal–fetal interface, initiated by the direct trophoblast injury provoked by aPL [21,22]. 

Yes-associated protein (YAP, also known as YAP1), a transcription co-activator, plays a crucial role in orchestrating the intricate programming of embryo growth and development [23,24,25]. Multifarious upstream intracellular signaling pathways, including Hippo, MAPK, and TLR [26,27,28], were demonstrated to mediate YAP protein activity on downstream target gene transcription, triggered by cell polarity, cell density, and energy status [29,30,31]. For example, when the Hippo signaling pathway is in the “on” state, sequential phosphorylation of upstream kinases phosphorylates YAP which is retained upon binding to 14-3-3 and then degraded in cytoplasm [30]. Unphosphorylated YAP is translocated into the nucleus and interacts with the transcriptional enhanced associate domain (TEAD) family of transcription factors to dually regulate target gene expression [24,32]. Verteporfin in vitro induced YAP cytoplasmic degradation in human embryonic stem cell-derived blastocyst-like spheroids greatly reduced the attachment rate and outgrowth area on receptive endometrium epithelial cells [23]. The accumulated phosphorylated YAP represses trophoblast cell stemness and cytokinesis, moving towards the development of early miscarriage, IUFGR and PE [33,34,35,36]. All that solid evidence seems to imply that excessive inactivation of YAP contributes to trophoblast dysfunction during placental early development and expansion. Nevertheless, detailed expression patterns of YAP and its specific roles in human trophoblasts exposed to aPL have not been revealed.

Taken together, we propose the hypothesis that YAP might act as a key effector integrating intracellular upstream signals triggered by aPL and modulating different trophoblast functions, a possible cellular mechanism to address the pathogenesis of pregnancy complications associated with aPL. Our study investigated the effect of circulating aPL from the serum of women with PM on YAP activity and cellular distribution in trophoblasts, and the biological role of YAP in trophoblast apoptosis, migration, and angiogenesis.

## 2. Materials and Methods

### 2.1. Antibody Preparation

Peripheral blood samples from aPL women carriers with pregnancy morbidity history and normal controls were collected in the anti-coagulant tubes. The study design was approved by the Ethics Committee of the Gynecology and Obstetrics Hospital, Fudan University (No.2021-70). The aPL women carriers were all identified with at least two consecutive aPL IgG positive results in hospital laboratory tests. Detailed information on the recruited individuals (normal control group, *n* = 6; aPL women carriers, *n* = 8) is provided in Supplemental Table S1. The serum was separated from the blood by centrifugation and purified with Melon™ Gel IgG Spin Purification Kit (Thermo Scientific, Rockford, IL, USA), following the manufacturer’s instructions. The purified polyclonal IgG was identified by ELISA (CUSABIO, Wuhan, China). After detecting the concentration, each IgG sample was diluted to 1 mg/mL and then sterilized through a 0.22 μm filter (Millipore, Darmstadt, Germany). The samples were stored at −80 °C for downstream application. 

### 2.2. Cell Culture and Treatments 

The human chorionic trophoblast cell line HTR-8/SVneo cells (referred as HTR-8 cells in the following text) were cultured in phenol red-free RPMI1640 (Gibco, Paisley, UK) supplemented with 10% fetal bovine serum (FBS, Gibco, Auckland, New Zealand) at 37 °C in a humid atmosphere with 5% CO_2_. When the cell confluency reached 80% on 6-well plates, cells were exposed to 100 µg/mL aPL negative (aPL−) or aPL positive (aPL+) IgG for 24 h (h). Cells were also exposed to mouse-IgG (M-IgG, 10 µg/mL, Beyotime, Shanghai, China)/bovine serum albumin (BSA, 100 µg/mL, Sigma) or anti-β2GPI-IgG (Sino-Biological, Beijing, China)/β2GPI (ProBio, Wuhan, China) at different concentrations [anti-β2GPI-IgG (0.1 µg/mL)/β2GPI (1 µg/mL) to anti-β2GPI-IgG (10 µg/mL)/β2GPI (100 µg/mL) with a binding ratio of 1:10] for 6 h with or without progesterone pretreatment for 24 h. Progesterone and RU486 (Sigma, Saint Louis, USA) were dissolved in dimethyl sulfoxide (DMSO, Sigma) respectively. Progesterone solution was added to the culture medium at different concentrations (10^−7^ to 10^−5^ M) alone or with progesterone antagonist RU486 (10^−5^ M). DMSO vehicle control was included. 

### 2.3. Plasmid and siRNA Transfections

YAP1 overexpression (OE) plasmid and YAP small interfering RNA (siRNA) were constructed by Genomeditech company (Shanghai, China). Full-length human YAP1 DNA (NM_001130145.2) was inserted into the PGMLV vector. The siRNA was designed against human YAP sequences: 5′-GACCAAUAGCUCAGAUCCUUUtt-3′. When HTR-8 cells were 70% confluent in 12-well plates, they were transfected with YAP1-OE plasmid (2 μg/well) or YAP siRNAs (30 pmol/well) using Lipofectamine 3000 transfection reagent (Invitrogen, Carlsbad, USA). The empty PGMLV vector or negative scrambled siRNA was used as controls, respectively. Gene expression was confirmed using RT-qPCR and immunoblotting. HTR-8 cells transfected with either the scrambled or YAP siRNAs were used for functional assays.

### 2.4. RNA Isolation, cDNA Synthesis, and Real-Time Quantitative PCR

The total RNA was extracted from HTR-8 cells through isolation with an EZ-press RNA purification Kit (EZBioscience, Roseville, USA). First-strand cDNA was then obtained with the Reverse Transcription Kit with gDNA Remover (EZBioscience). Real-time quantitative PCR was performed using SYBR Green qPCR Master Mix (ROX1 plus, EZBioscience) and the ABI 7900 system (Applied Biosystems, Foster, USA). The samples were run in triplicate and comparative cycle threshold method was used to calculate relative mRNA expression normalized to GAPDH (Table 1).

### 2.5. Immunoblotting

All cells were lysed in radioimmunoprecipitation assay (RIPA) buffer plus 1% phosphatase inhibitors (New Cell & Molecular Biotech, Suzhou, China). All lysates were quantitated with a BCA Assay and mixed with 5× SDS loading buffer and boiled for 10 min. Equal amounts of total protein were loaded into each lane of a 10% polyacrylamide gel and separated by electrophoresis, followed by transfer onto a polyvinylidene fluoride (PVDF) membrane (Invitrogen). Membranes were blocked in 5% nonfat dry milk/Tris-buffered saline-0.5% Tween 20 and then incubated with primary antibodies overnight at 4 °C. GAPDH was used as a loading control. The membranes were further probed with horseradish-peroxidase-conjugated secondary antibody for 1 h at room temperature. The protein levels were detected by using the enhanced chemiluminescence (ECL) Immunoblot Analysis Detection System.

The primary antibodies used were as following: YAP (ab52771, Abcam, Cambridge, UK); phospho-YAP (ser 127) (ab76252, Abcam); ERK1/2 (#4695, CST, Danvers, MA, USA); phospho-ERK1/2 (#4370, CST); p38 MAPK (#8690, CST); phospho-p38 MAPK (#4511, CST); BAX (50599-2-Ig, Proteintech, Rosemont, USA); BCL2 (12789-1-AP, Proteintech); and VEGF (19003-1-AP, Proteintech).

### 2.6. Immunofluorescence Staining

HTR-8 cells were cultured on glass coverslips and treated with M-IgG/BSA or anti-β2GPI-IgG/β2GPI with or without progesterone pretreatment for 6 h at 37 °C. After being fixed with 4% paraformaldehyde and permeabilized with 0.2% Triton X-100, cells were blocked with 5% goat serum for 2 h at room temperature and subsequently incubated with 488-conjugated YAP antibody (1:100, Proteintech) overnight at 4 °C. The nuclei were stained with 4′-6-diamidino-2-phenylindole (DAPI, Beyotime) for 8 min. The images were captured by inverted fluorescence microscope (Olympus, Tokyo, Japan).

### 2.7. Cell Apoptosis and Cell Cycle Assays

For the cell apoptosis assay, HTR-8 cells transfected with YAP siRNA after 48 h were detected by Annexin V-FITC Apoptosis Detection Kit (Dojindo, Kumamoto, Japan) following the manufacturer’s instructions. Cells stained with FITC and propidium iodide (PI) solutions were analyzed by flow cytometer. For the cell cycle assay, the HTR-8 cells were collected after transfection with YAP siRNA for 48 h and then fixed by ethanol. The cell cycle distribution of fixed cells stained by using Cell Cycle Staining Kit (Multi Sciences, Hangzhou, China) was determined by flow cytometry. Both assays were carried out in triplicate. Flow cytometry data were plotted and quantified with FlowJo software (version 10; Ashland, OR, USA). 

### 2.8. Cell Migration Assay

Cell migration ability was determined by using wound-healing assay. HTR-8 cells were seeded at 1 × 10^5^ cells on 12-well plates. After transfection with YAP siRNA for 24 h, the plates were scratched with a 200 µL pipette tip and the culture medium was replaced with RPMI1640 containing 1% FBS. The scratch areas in the same fields per well were recorded under a light microscope (Olympus) at 0 h, 20 h, and 30 h. The wound recovery rate was calculated as the scratch areas at different time points over the initial scratch areas (0 h) in percentages. 

### 2.9. Tube Formation Assay

A 50 µL/well of cold Matrigel (Corning, Bedford, USA) was added to pre-cooled 96-well plates and then incubated at 37 °C for 30 min until Matrigel solidified. After HTR-8 cells were transfected with YAP siRNA or scrambled siRNA for 48 h, the medium was replaced with fresh culture medium and collected after another 24 h. Human umbilical vein endothelial cells (HUVEC) were resuspended in the collected conditioned culture medium and seeded on the Matrigel-coated plates (2000 cells/well). Tubules formed after 3 h in the cell culture incubator and were observed under inverted microscope. The digital images were analyzed by Image J angiogenesis analyzer.

### 2.10. Statistical Analysis

All data are presented as the mean ± SEM. The significance of the results was assessed by Student’s *t* test, one-way ANOVA test, or Mann–Whitney U test using the GraphPad Prism software package (version 8.0; La Jolla, USA); *p* < 0.05 was considered to be significant.

## 3. Results

### 3.1. aPL+ IgG Affects YAP Expression but Does Not Promote the Phosphorylation of YAP Protein in HTR-8 Cells

**Figure 1:**
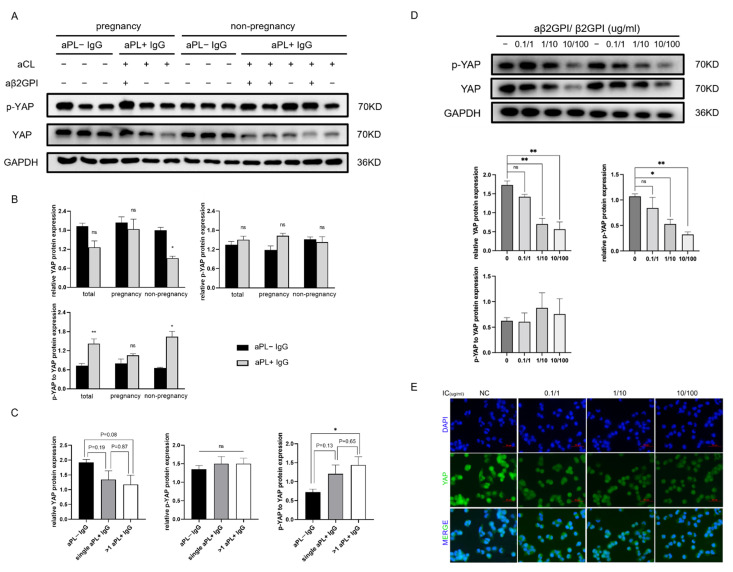
aPL-positive IgG affects YAP expression and nuclear localization in HTR-8 cells. aPL+ IgG was extracted from serum aPL-positive women carriers with pregnancy morbidity history and aPL− IgG from normal women for comparison. (**A**) YAP and phospho-Ser 127 YAP in HTR-8 cells treated with aPL− IgG (100 μg/mL) and aPL+ IgG (100 μg/mL) for 24 h were immunoblotted. Subgroup analysis was performed based on pregnancy status (**B**) and single or more aPL+ IgG (**C**). Single aPL+ IgG indicated that only aCL IgG or anti-β2GPI IgG was positive. More than one aPL+ IgG indicated both aCL IgG and anti-β2GPI IgG positivity. The anti-β2GPI-IgG monoclonal antibody and human β2GPI (anti-β2GPI-IgG/β2GPI) complex was also prepared. (**D**) The anti-β2GPI-IgG/β2GPI complex suppressed YAP expression of HTR-8 cells in a dose-dependent manner, with concentrations ranging from 0.1/1 to 10/100 μg/mL with a binding ratio of 1:10. Quantitation of total YAP and p-YAP levels from blots (normalized to GAPDH) are shown as the mean± SEM (**B**–**D**). Mann–Whitney U test; ns indicates no significance, * *p* < 0.05, ** *p* < 0.01. (**E**) Incubation with the anti-β2GPI-IgG/β2GPI complex for 6 h caused cytoplasmic retention of YAP in HTR-8 cells. The cellular distribution of YAP was shown with representative immunostaining images of HTR-8 cells after 6 h of exposure to different concentrations of anti-β2GPI-IgG/β2GPI. Scale bars, 50 μm. NC, control; IC, anti-β2GPI-IgG/β2GPI complex.

To investigate the aPL-induced variation of YAP protein in the trophoblast compared to the normal one, we treated human trophoblast cell lines HTR-8/SVneo with polyclonal aPL+ IgG from aPL women carriers or aPL− IgG from normal controls for 24 h. Furthermore, the expression of YAP and phosphorylated YAP (p-YAP) protein in human trophoblast cell lines was determined by immunoblotting (Figure 1A). YAP expression was evidently decreased between the aPL+ IgG group and the aPL− IgG group (*p* < 0.01), especially in non-pregnant women (*p* < 0.05) (Figure 1B). It is worth noting that YAP levels in the obstetric aPL+ IgG group remained without significance change. Phosphorylation of YAP promotes cytoplasmic retention and culminates in degradation [37]. The expected increase in p-YAP levels induced by aPL+ IgG was not observed (*p* > 0.05). In the obstetric aPL+ IgG group, there was merely a slight uptrend in the p-YAP level without significance. Downregulation of YAP expression in HTR-8 cells after aPL+ IgG treatment might be through other potential molecular mechanisms, not only subsequent to phosphorylation. Additionally, we analyzed the alteration of YAP expression in HTR-8 cells treated with single or double positive aPL subsets (Figure 1C). A declining trend of YAP level in HTR-8 cells was presented with double aCL IgG and anti-β2GPI IgG positivity (*p* = 0.08). The p-YAP/YAP ratio was significantly increased between the >1 aPL+ IgG group and the normal control group (*p* < 0.05), but not the single aPL+ IgG group vs. the normal control group, suggesting that the number of aPL IgG subsets may affect YAP activity. 

### 3.2. Anti-β2GPI-IgG Monoclonal Antibody and Human β2GPI Complex Function in a Dose-Dependent Manner to Reduce YAP Expression and Nuclear Localization in HTR-8 Cells

To determine whether the variation of YAP affected by aPL+ IgG is indeed caused by antiphospholipid antibodies, we used anti-β2GPI-IgG monoclonal antibody combined with β2GPI (anti-β2GPI-IgG/β2GPI complex) to mimic the placenta-site damage of aPL. The anti-β2GPI antibody is well proven to target endogenous β2GPI upon trophoblasts, considered as the primary antigen in APS, with good specificity in prediction for pregnancy morbidity. The relative level of YAP protein in HTR-8 cells exposed to anti-β2GPI-IgG (1 µg/mL)/β2GPI (10 µg/mL) complex for 6 h significantly decreased compared to isotype IgG (1 µg/mL)/BSA (10 µg/mL) control (*p* < 0.01). The administration of the anti-β2GPI-IgG/β2GPI complex at different concentrations [anti-β2GPI-IgG/β2GPI, from 0.1/1 (µg/mL) to 10/100 (µg/mL), with a binding ratio of 1:10] inhibited YAP protein expression in a dose-dependent manner, with maximal inhibition at anti-β2GPI-IgG (10 µg/mL)/β2GPI (100 µg/mL) (Figure 1D). In parallel, YAP protein was restored in cytoplasm upon treatment with the anti-β2GPI-IgG/β2GPI complex (Figure 1E). These results implied that the anti-β2GPI-IgG/β2GPI complex shows a dose–response effect on YAP downregulation and inhibits YAP nuclear translocation in HTR-8 cells.

### 3.3. Progesterone Enhances the Expression of YAP and Is Partly Reversed by RU486 in HTR-8 Cells

**Figure 2:**
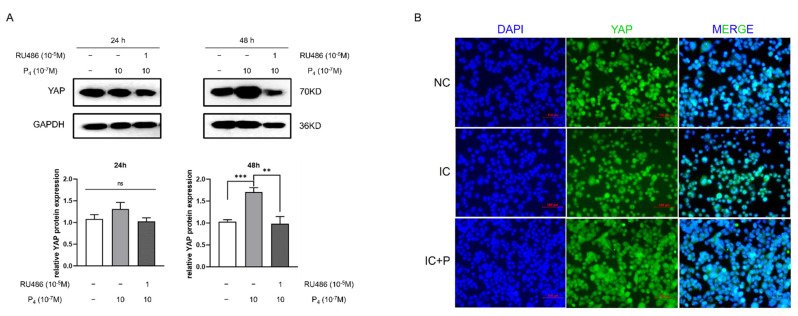
Progesterone regulates the expression of YAP in HTR-8 cells. (**A**) Progesterone enhances the expression of YAP and is partly reversed by RU486 in HTR-8 cells. HTR-8 cells were treated with control vehicle (DMSO) or progesterone (P_4_) (10^−6^ M), alone or in combination with RU486 (10^−5^ M), for 24 h or 48 h. The YAP protein expression in each group was analyzed by immunoblotting and GAPDH served as internal control. The results of quantification are presented as the mean ± SEM. One-way ANOVA test; ns indicates nonsignificant, ** *p* < 0.01, *** *p* < 0.001. (**B**) Pre-treatment of P_4_ (10^−6^ M) for 24 h promotes nuclear translocation of YAP in HTR-8 cells administrated by anti-β2GPI-IgG (1 μg/mL)/β2GPI (10 μg/mL). Scale bars, 100 μm. NC, control; IC, the anti-β2GPI-IgG/β2GPI complex; IC+P, the anti-β2GPI-IgG/β2GPI complex with progesterone pretreatment.

Considering the difference of YAP regulation between pregnant women with APS and non-pregnant women with APS, we discovered the effect of hormones associated with pregnancy on YAP expression. To determine whether progesterone has the same behavior in the trophoblast, we further applied progesterone at certain gradient concentrations (10^−7^ M to 10^−5^ M) to culture medium to detect the alteration of YAP expression after 24 h (Figure S1). However, there were no significant changes. When progesterone treatment was extended for another 24 h, the expression of YAP protein was promoted at 10^−6^ M (*p* < 0.05) (Figure 2A). Blocking the progesterone receptor with its inhibitor RU486 compromised the stimulatory effect of progesterone on YAP expression. To further verify the protective action of progesterone on YAP expression, we pre-incubated HTR-8 cells with progesterone for 24 h before the addition of the anti-β2GPI-IgG/β2GPI complex. Immunostaining results showed that, after HTR-8 cells were treated with anti-β2GPI-IgG/β2GPI complex for 6 h, the 10^−6^ M progesterone pretreatment group showed relatively reduced cytoplasmic retention of YAP compared with the control group (Figure 2B). It is suggesting that progesterone might partly rescue the reduction of YAP affected by anti-β2GPI-IgG/β2GPI complex and promote YAP translocation into the nucleus of HTR-8 cells.

### 3.4. YAP Depletion Induces Cell Cycle Arrest and Cell Apoptosis of Trophoblast

**Figure 3:**
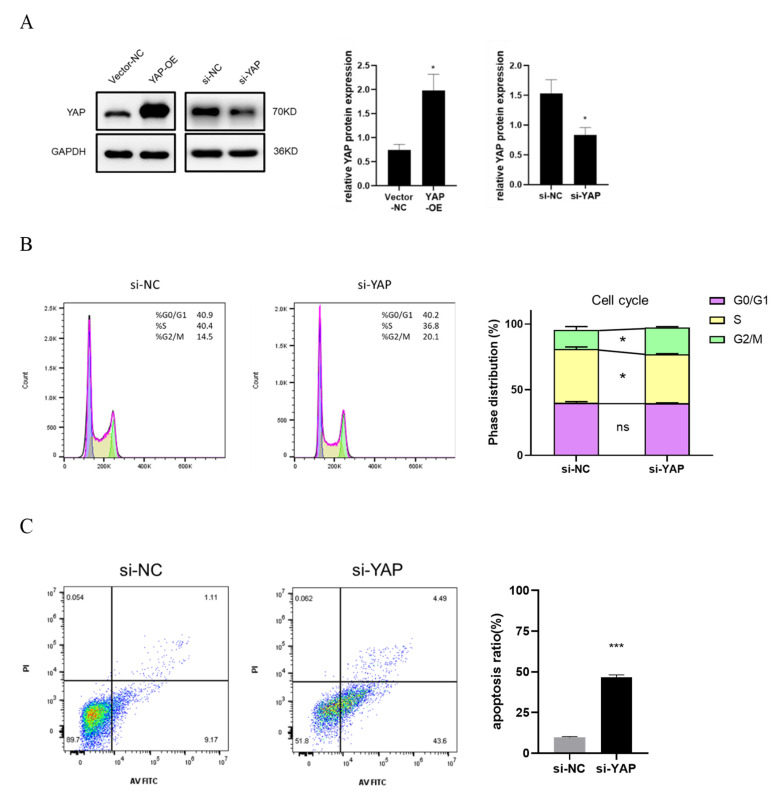

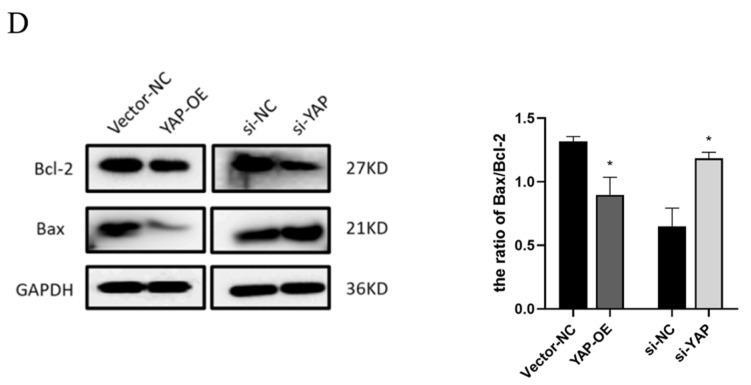
Downregulated YAP induces cell apoptosis and blocks cell cycle progression. (**A**) Confirmation of YAP overexpression and knockdown in HTR-8 cells after transfection with YAP-OE plasmid (YAP-OE) or YAP siRNA (si-YAP) by immunoblotting. Vector plasmid and control siRNA served as controls. (**B**) Analysis of cell cycle in HTR-8 cells transfected with YAP siRNA transfection was performed using flow cytometer. The si-NC group served as the control. The phases of G0/G1, S, and G2/M were calculated. (**C**) The percentage of apoptotic cells in si-YAP HTR-8 cells was determined by using flow cytometer. (**D**) Immunoblotting of Bax and Bcl2 protein levels in HTR-8 cells transfected with YAP-OE plasmid or YAP siRNA. The ratio of Bax and Bcl2 protein level was quantified. All results are shown as mean ± SEM. Student’s *t* test; * *p* < 0.05, *** *p* < 0.001.

To directly detect the role of YAP in the trophoblast cell cycle and apoptosis, HTR-8 cells transfected with YAP siRNA (si-YAP) and compared with those transfected with scrambled siRNA (si-NC) for 48 h (Figure 3A) were collected for flow cytometry analysis. After YAP knockdown, the cell cycle of HTR-8 was stopped at the G2/M phase (*p* < 0.05) (Figure 3B). The proportion of HTR-8 cells at the S phase (DNA synthesis period) was significantly reduced (*p* < 0.05). Furthermore, we found that the apoptosis rate of si-YAP HTR-8 cells was increased by 36.9% in contrast to that of the control (*p* < 0.001) (Figure 3C). YAP was also overexpressed in HTR-8 cells (YAP-OE) by transfection with PGMLV-YAP1 plasmid. The immunoblotting results showed that the absence of YAP led to a higher ratio of pro-apoptotic protein Bax to anti-apoptotic protein Bcl2, while the result of YAP overexpression was the opposite (Figure 3D). These results indicated that YAP depletion of HTR-8 cells interfered with cell cycle distribution and induced cell apoptosis dependently of Bax/Bcl2 ratio.

### 3.5. Downregulated YAP in Trophoblast Impairs Cell Migration and Tube Formation

**Figure 4:**
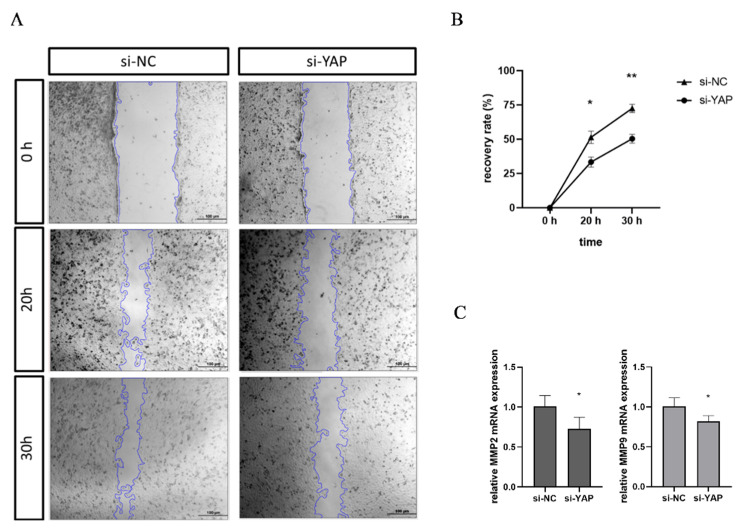
YAP knockdown impairs HTR-8 cell migration ability. (**A**) The wound healing assay was conducted between si-NC HTR-8 and si-YAP HTR-8 cells groups and the representative images were taken at 0 h, 20 h, and 30 h. Scale bars are 100 µm. (**B**) The recovery rates of HTR-8 cells from the two groups are summarized. (**C**) The mRNA levels of MMP2 and MMP9 in HTR-8 cells transfected with control siRNA or YAP siRNA for 48 h were determined by RT-qPCR. Results are shown as mean ± SEM. Student’s *t* test; * *p* < 0.05, ** *p* < 0.01.

We studied the effect of YAP protein on the ability of trophoblast migration in vitro by wound healing assay 48 h after knockdown of YAP by siRNA. As shown in Figure 4A,B, the scratch recovery rate was significantly descending in the si-YAP group along with the si-NC group. Over 20 h and 30 h, the migration of si-YAP HTR-8 cell was inhibited by 18.9% (*p* < 0.05) and 22.2% (*p* < 0.01), respectively. The mRNA levels of MMP2 and MMP9 were decreased in cells depleted of YAP (Figure 4C), but they were not seen significantly changed under YAP overexpression (Figure S2). 

**Figure 5:**
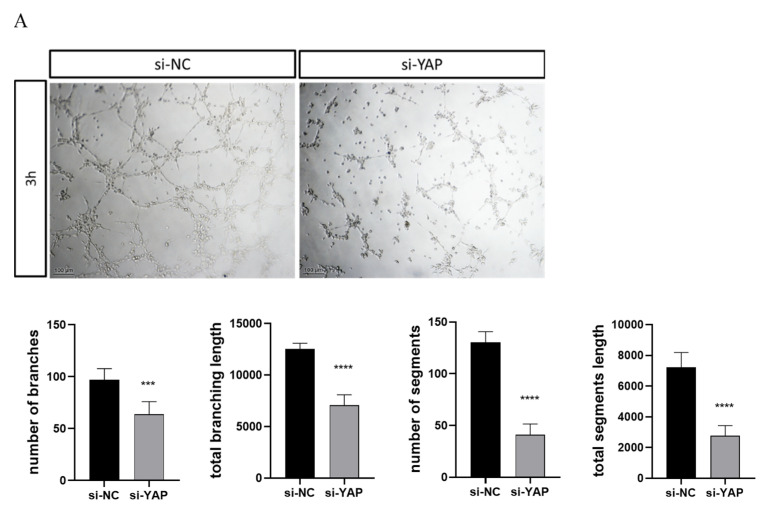

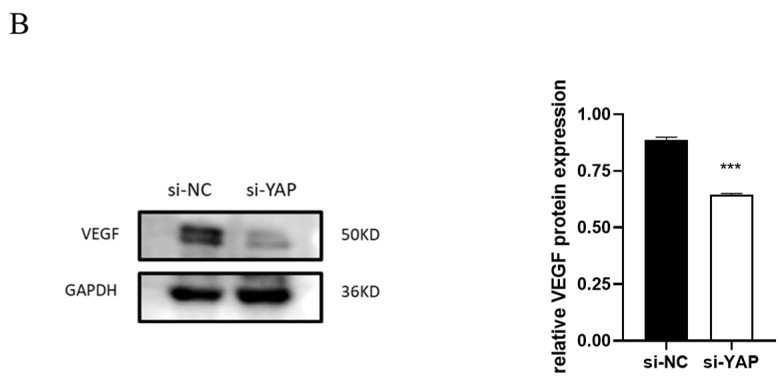
YAP-mediated VEGF expression from HTR-8 cell is essential for tube forming capability of HUVECs. HUVECs were seeded onto Matrigel and incubated with culture supernatant from HTR-8 cells transfected with scrambled siRNA or YAP siRNA. (**A**) The tube formation assay was analyzed after 3 h. Representative images are shown. Scale bars are 100 µm. (**B**) The VEGF protein expression in HTR-8 cells 72 h after transfection with scrambled siRNA and YAP siRNA was measured by immunoblotting (normalized to GAPDH). Results are shown as mean ± SEM. Student’s *t* test; *** *p* < 0.001, **** *p* < 0.0001).

The supernatant of cell culture medium 72 h later was additionally harvested for co-culture with HUVECs to assess the tube formation ability of the trophoblast. Four indices showed a magnificent decrease in HUVECs cocultured with the culture medium supernatant from the si-YAP group (the number of branches by 34%, the total branching length by 44%, the number of segments by 69%, the total segments length by 62%) (Figure 5A). Based on the immunoblotting results, the expression of VEGF (vascular endothelial growth factor) in YAP siRNA-treated cells was largely downregulated (Figure 5B). Thus, YAP aberration attenuated the ability of trophoblast migration and tube formation, which was necessary for spiral artery remodeling in the placenta.

### 3.6. YAP Knockdown Results in the Activation of ERK1/2 in HTR-8 Cells

**Figure 6:**
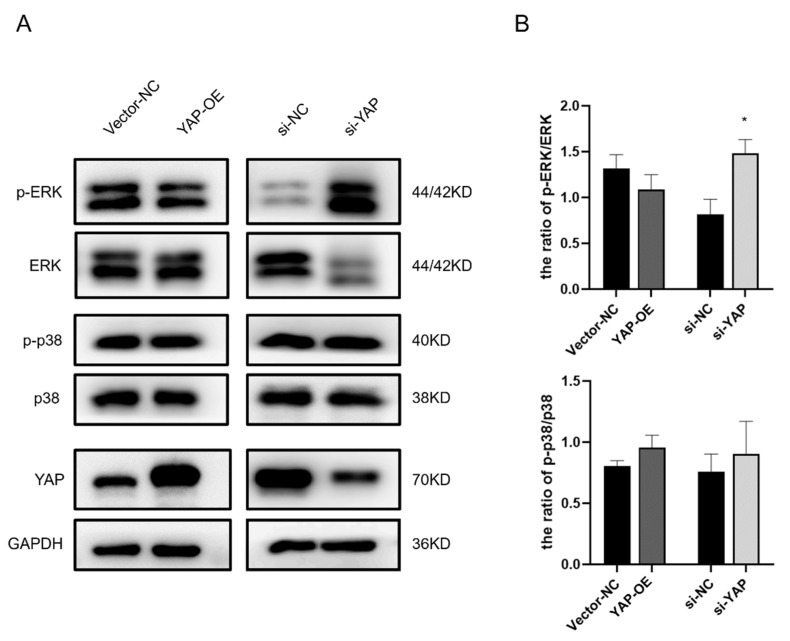
The phosphorylation of MAPK-ERK ascends after YAP inhibition in HTR-8 cells. YAP-OE plasmid (YAP-OE) or YAP siRNA (si-YAP) were transfected into HTR-8 cells which were harvested after 72 h. (**A**) Immunoblotting of the p-ERK, ERK, p-p38, and p38 proteins in YAP-OE or si-YAP HTR-8 cells. (**B**) p-ERK and p-p38 protein levels were normalized to ERK or p38 protein levels. GAPDH served as internal control. All values are presented as mean ± SEM. Student’s *t* test; * *p* < 0.05.

The MAP kinase (MAPK) signaling pathway was proven to be one of the critical links between YAP activation and its downstream target gene expression in mammalian cells. In addition, earlier research showed that ERK (MAPK family) inhibition treatment induced YAP inactivation in the trophoblast [34]. To further study the underlying association between YAP protein and MAPK activation in the trophoblast, we examined the relative Thr202/Tyr204 phosphorylation of ERK1/2 protein level and the relative Thr180/Tyr182 phosphorylation of p38 protein level which are indicative of their activation (Figure 6A). In HTR-8 cells with YAP silencing, ERK1/2 activation was stimulated significantly, but this effect was not observed in HTR-8 cells overexpressing YAP (Figure 6B). In contrast, neither YAP overexpression nor knockdown inhibited or activated p38. 

## 4. Discussion

Our work uncovered a strikingly new discovery that aPL induced a decrease in YAP protein expression in human trophoblast cell lines. Anti-β2GPI-IgG binding to β2GPI functions in a dose-dependent manner to reduce YAP expression and causes a corresponding cytoplasm YAP translocation in HTR-8 cells. The aberrant downregulation of YAP will display a reduction in cellular functional output—cell cycle, apoptosis, migration, and angiogenesis. These findings about YAP suggest a potentially critical mechanistic link between aPL stimulus and alterations in trophoblast function.

YAP, like an “on-and-off gate” transcription co-activator, defines the cell fate decision in trophectoderm lineage specification during preimplantation [38] and guides trophoblast differentiation throughout the early placenta development [39]. Our in vitro experiments identified that downregulating YAP in HTR-8 cells augments cell apoptosis via raising the ratio of Bax/Bcl2 and blocks the cell cycle in the G2/M phase leading to cell growth arrest. In cytotrophoblast progenitors, YAP fully binding with TEAD4 manipulates the transcription of cell-cycle regulators, including CDK1 and CYCLINs, to maintain the self-renewal of the trophoblast [32]. Similarly, CDK1 and CCNB1, controlling the G2/M phase transition, are enriched in the most proliferative CTB subset in the first-trimester human placenta by a single-cell RNA-seq analysis [40]. Loss of YAP in the trophoblast may affect cell-cycle-restricted gene expression in the process of cell stemness and cell–cell fusion [33]. Rapid activation and maintenance of a robust YAP transcriptional program is necessary for trophoblast population expansion and functional human placental syncytia. Moreover, trophoblast invasion and migration were attenuated through unexplained activation of the Hippo-YAP1 signaling pathway [35,36]. Consistent with this, we further revealed that YAP depletion impaired the transcription of proteolytic enzymes MMP2/9 and secretion of the angiogenic factor VEGF in the trophoblast. MMPs and VEGF are critical cytokines involved in invasive trophoblast replacement of the uterine spiral artery endothelium in the process of placentation. Unsuccessful placentation and abnormal placental function in APS patients result in insufficient blood supply to the fetus in the uterus, ultimately increasing the incidence of early-onset pre-eclampsia, IUGR, and preterm labor. 

The Hippo-signaling-dependent coactivator YAP activity control determines human and murine trophoblast maintenance and expansion [41,42]. Activation of the Hippo signaling pathway encourages the engagement of the core phosphorylated kinase cassette, regulated by interplay and feedback with other signaling such as Wnt and Notch signaling during early placental development, allowing for context-specific responses [43,44]. We first discovered that aPL+ IgG attenuated YAP expression in HTR-8 cells, rather than accelerated the phosphorylation of YAP. Considering the anti-β2GPI antibody, among all subtypes of anti-phospholipid antibodies, is one of the most frequently used prognostic indices for pregnancy morbidity in APS patients [14], we further found that the aβ2GPI-IgG/β2GPI complex reveals dose-dependent characteristics in reducing YAP expression and nuclear localization. It is not yet known whether the anti-β2GPI-IgG inhibition on YAP relies on a phosphorylated kinase cassette or direct abrogation of YAP gene expression, and whether this impact on YAP is widespread in the trophoblast stimulated by other criteria than aPL, such as aCL and LA. Our current understanding of the principle of YAP degradation is merely limited to subsequent phosphorylated kinase cascade. The molecular mechanism employed appears uncertain, indicating a promising study on how exactly aPL work on YAP1 protein downregulation or degradation during placental dysfunction.

The aPL binding to β2GPI on the trophoblast surface inhibits invasion through MAPK [18]. In human IUFGR placenta, ERK/MAPK inhibition acts on YAP phosphorylation as an upstream signaling pathway [34]. MAPK-ERK and YAP were demonstrated to share similar effects on cell apoptosis, proliferation, and oncogenesis in different cancer cells [26]. To determine the correlation between YAP and ERK, we knocked down YAP expression in the trophoblast and further found that YAP negatively regulates ERK activation in turn. MAPK/ERK signaling influences the expression of downstream genes such as MMPs and cadherins in the regulation of trophoblast cell invasion and migration [45]. We consider that there is a negative feedback mechanism between YAP activation and ERK phosphorylation in the trophoblast that helps rescue cellular homeostasis against the external disturbance. It will be interesting to explore whether ERK activation occurred in response to balance YAP downregulation in aPL-treated trophoblasts and the consequent outcomes when both ERK and YAP pathways are inactivated.

In this study, we observed that aPL from these obstetric aPL carriers neither significantly induced the decline of YAP nor accelerated the phosphorylation of YAP in the trophoblast. Could this variation of YAP come from the protective support of pregnancy-related hormones at the maternal–fetal interface after medicine intervention? 

Progesterone, a hormone critical to pregnancy protection, is produced by the corpus luteum and the placenta after the corpus luteum regression [46]. It promotes the endometrial decidualization, modulates mammalian maternal–fetal immune tolerance and decreases the resistance of the spiral arteries to maintain a successful gestation [47,48]. In an APS mouse model, progesterone supplementation inhibits complement-activation-mediated thrombosis and inflammatory injury in the placenta and avoids spontaneous pregnancy loss [49]. YAP has been proved to be a progesterone-responsive gene in fetal mouse cardiomyocytes [50]. Recent research in ovariectomy mice also explored whether progesterone support in estrogen deprivation induced the formation of YAP-TEAD4 complex contributing to CDX2-mediated trophectoderm differentiation during peri-implantation [51]. Our results revealed that a high physiological concentration of progesterone in pregnancy facilitates YAP expression of human trophoblast cell line HTR-8 in a progesterone-receptor-dependent manner. The enhancing effect of progesterone on YAP expression promotes YAP nuclear translocation which is attenuated by aPL. It is indicated that progesterone might reverse the negative effect of aPL on YAP activity. Studies of progesterone action on YAP expression in the trophoblast in the presence of aPL or not in vivo are now warranted. Furthermore, more research groups have started to propose alternative treatment approaches in addition to the traditional routine therapy of low molecular weight heparin alone, or in combination with low-dose aspirin or hydroxychloroquine treatment. The promising alternative treatment options include but are not limited to hydroxychloroquine [52], aspirin-triggered lipoxin [53], progesterone [49], and ApoER2 gene knockout [15]. Apart from aPL-mediated PM, the blockade of YAP nuclear translocation has been reported in other pregnancy complications owing to poor placentation such as early pregnancy loss, PE, and IUFGR. Our findings provide a firm research basis for YAP serving as a potential therapeutic target in the prevention of pregnancy morbidity in future.

## 5. Conclusions

**Figure 7:**
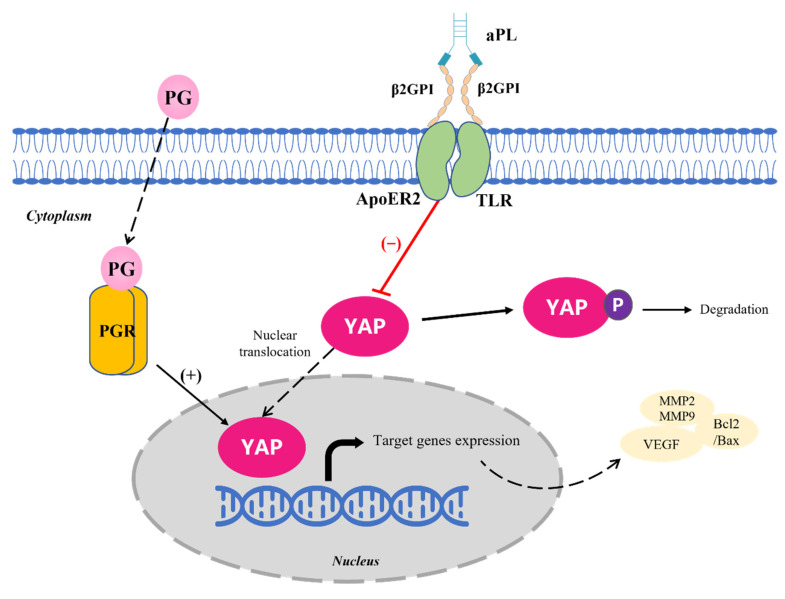
Schematic diagram of the hypothesized molecular mechanism for YAP protein involvement in aPL-induced pregnancy complications. aPL was previously demonstrated to recognize β2GPI on the trophoblast surface and provoke intra-cellular response dependent upon Toll-like receptor (TLR) or LDL receptor family member apoE receptor 2 (ApoER2) [15,17]. We assume that aPL interacting with β2GPI-ApoER2 or β2GPI-TLR on the trophoblast induces aberrant inactivation of YAP protein and thereafter impacts the expression of cell apoptosis, migration, and angiogenesis genes, which can be rescued by progesterone.

In summary, treatment of the trophoblast with aPL+ IgG from non-obstetric clinical donors with pregnancy morbidity induced YAP inactivation. Moreover, the monoclonal aβ2GPI-IgG together with β2GPI administration confirmed the dose-dependent effect on decrease of YAP expression and nuclear translocation. Knocking out the YAP gene disturbs the normal trophoblast cell function involved with cell cycle, apoptosis, migration, and angiogenesis. A high physiological concentration of progesterone in pregnancy facilitates YAP expression in the trophoblast. We propose that the attack of aPL blocks the nuclear translocation and activity of YAP, resulting in consequent trophoblast dysfunction and progesterone may rescue YAP expression in maintaining pregnancy against the impact of aPL (Figure 7). 

## Figures and Tables

**Table 1:** Real-time quantitative PCR primer sequences used in this study.

	Sequence5′-3′
YAP-F	ATGAACTCGGCTTCAGGTC
YAP-R	AGCCAAGAGGTGGTCTTGTT
MMP2-F	GATACCCCTTTGACGGTAAGGA
MMP2-R	CCTTCTCCCAAGGTCCATAGC
MMP9-F	AGACCTGGGCAGATTCCAAAC
MMP9-R	CGGCAAGTCTTCCGAGTAGT
GAPDH-F	GGAGCGAGATCCCTCCAAAAT
GAPDH-R	GGCTGTTGTCATACTTCTCATGG

## Data Availability

The data presented in this study are available on request from the corresponding author.

## Supplementary Materials

The following supporting information can be downloaded at: https://www.mdpi.com/article/10.3390/biomedicines10123296/s1, Table S1: Clinical and laboratory features of healthy women and aPL women carriers; Figure S1: Progesterone treatment for 24 h has no effect on YAP protein expression in vitro; Figure S2: YAP overexpression does not alter the mRNA expression of MMP2 or MMP9.

## References

[1] Nalli C., Somma V., Andreoli L., Buttner T., Schierack P., Mahler M., Roggenbuck D., Tincani A. (2018). Anti-Phospholipid IgG Antibodies Detected by Line Immunoassay Differentiate Patients with Anti-Phospholipid Syndrome and Other Autoimmune Diseases. Auto Immun. Highlights.

[2] Saccone G., Berghella V., Maruotti G.M., Ghi T., Rizzo G., Simonazzi G., Rizzo N., Facchinetti F., Dall’Asta A., Visentin S. (2017). Antiphospholipid Antibody Profile Based Obstetric Outcomes of Primary Antiphospholipid Syndrome: The PREGNANTS Study. Am. J. Obs. Gynecol..

[3] Garcia D., Erkan D. (2018). Diagnosis and Management of the Antiphospholipid Syndrome. N. Engl. J. Med..

[4] Clark E.A., Silver R.M., Branch D.W. (2007). Do Antiphospholipid Antibodies Cause Preeclampsia and HELLP Syndrome?. Curr. Rheumatol. Rep..

[5] Santos T.D.S., Ieque A.L., de Carvalho H.C., Sell A.M., Lonardoni M.V.C., Demarchi I.G., de Lima Neto Q.A., Teixeira J.J.V. (2017). Antiphospholipid Syndrome and Recurrent Miscarriage: A Systematic Review and Meta-Analysis. J. Reprod. Immunol..

[6] Opatrny L., David M., Kahn S.R., Shrier I., Rey E. (2006). Association between Antiphospholipid Antibodies and Recurrent Fetal Loss in Women without Autoimmune Disease: A Metaanalysis. J. Rheumatol..

[7] Ofer-Shiber S., Molad Y. (2015). Frequency of Vascular and Pregnancy Morbidity in Patients with Low vs. Moderate-to-High Titers of Antiphospholipid Antibodies. Blood Coagul. Fibrinolysis.

[8] De Carolis S., Tabacco S., Rizzo F., Giannini A., Botta A., Salvi S., Garufi C., Panici P.B., Lanzone A. (2018). Antiphospholipid Syndrome: An Update on Risk Factors for Pregnancy Outcome. Autoimmun. Rev..

[9] Viall C.A., Chamley L.W. (2015). Histopathology in the Placentae of Women with Antiphospholipid Antibodies: A Systematic Review of the Literature. Autoimmun. Rev..

[10] Ripoll V.M., Pregnolato F., Mazza S., Bodio C., Grossi C., McDonnell T., Pericleous C., Meroni P.L., Isenberg D.A., Rahman A. (2018). Gene Expression Profiling Identifies Distinct Molecular Signatures in Thrombotic and Obstetric Antiphospholipid Syndrome. J. Autoimmun..

[11] Poulton K., Ripoll V.M., Pericleous C., Meroni P.L., Gerosa M., Ioannou Y., Rahman A., Giles I.P. (2015). Purified IgG from Patients with Obstetric but Not IgG from Non-Obstetric Antiphospholipid Syndrome Inhibit Trophoblast Invasion. Am. J. Reprod. Immunol..

[12] Liu T., Han J., Zhang R., Tang Z., Yi G., Gong W., Wan L., Hu Q., Teng J., Liu H. (2022). Characteristics of Purified Anti-Beta2GPI IgG N-Glycosylation Associate with Thrombotic, Obstetric and Catastrophic Antiphospholipid Syndrome. Rheumatology (Oxford).

[13] Chaturvedi S., McCrae K.R. (2017). Diagnosis and Management of the Antiphospholipid Syndrome. Blood Rev..

[14] Fierro J.J., Velasquez M., Cadavid A.P., de Leeuw K. (2021). Effects of Anti-Beta 2-Glycoprotein 1 Antibodies and Its Association with Pregnancy-Related Morbidity in Antiphospholipid Syndrome. Am. J. Reprod. Immunol..

[15] Ulrich V., Gelber S.E., Vukelic M., Sacharidou A., Herz J., Urbanus R.T., de Groot P.G., Natale D.R., Harihara A., Redecha P. (2016). ApoE Receptor 2 Mediation of Trophoblast Dysfunction and Pregnancy Complications Induced by Antiphospholipid Antibodies in Mice. Arthritis Rheumatol..

[16] Gysler S.M., Mulla M.J., Guerra M., Brosens J.J., Salmon J.E., Chamley L.W., Abrahams V.M. (2016). Antiphospholipid Antibody-Induced MiR-146a-3p Drives Trophoblast Interleukin-8 Secretion through Activation of Toll-like Receptor 8. Mol. Hum. Reprod..

[17] Mulla M.J., Weel I.C., Potter J.A., Gysler S.M., Salmon J.E., Peracoli M.T.S., Rothlin C.V., Chamley L.W., Abrahams V.M. (2018). Antiphospholipid Antibodies Inhibit Trophoblast Toll-Like Receptor and Inflammasome Negative Regulators. Arthritis Rheumatol..

[18] Krivokuća M.J., Abu Rabi T., Stefanoska I., Vrzić-Petronijević S., Petronijević M., Vićovac L. (2017). Immunoglobulins from sera of APS patients bind HTR-8/SVneo trophoblast cell line and reduce additional mediators of cell invasion. Reprod. Biol..

[19] Marchetti T., Ruffatti A., Wuillemin C., de Moerloose P., Cohen M. (2014). Hydroxychloroquine Restores Trophoblast Fusion Affected by Antiphospholipid Antibodies. J. Thromb. Haemost..

[20] Di Simone N., D’Ippolito S., Marana R., di Nicuolo F., Castellani R., Pierangeli S.S., Chen P., Tersigni C., Scambia G., Meroni P.L. (2013). Antiphospholipid Antibodies Affect Human Endometrial Angiogenesis: Protective Effect of a Synthetic Peptide (TIFI) Mimicking the Phospholipid Binding Site of Beta(2) Glycoprotein I. Am. J. Reprod. Immunol..

[21] Tong M., Viall C.A., Chamley L.W. (2015). Antiphospholipid Antibodies and the Placenta: A Systematic Review of Their in Vitro Effects and Modulation by Treatment. Hum. Reprod. Update.

[22] Meroni P.L., Borghi M.O., Raschi E., Tedesco F. (2011). Pathogenesis of Antiphospholipid Syndrome: Understanding the Antibodies. Nat. Rev. Rheumatol..

[23] Yue C., Chen A.C.H., Tian S., Fong S.W., Lee K.C., Zhang J., Ng E.H.Y., Lee K.F., Yeung W.S.B., Lee Y.L. (2020). Human Embryonic Stem Cell-Derived Blastocyst-like Spheroids Resemble Human Trophectoderm during Early Implantation Process. Fertil. Steril..

[24] Nishioka N., Inoue K., Adachi K., Kiyonari H., Ota M., Ralston A., Yabuta N., Hirahara S., Stephenson R.O., Ogonuki N. (2009). The Hippo Signaling Pathway Components Lats and Yap Pattern Tead4 Activity to Distinguish Mouse Trophectoderm from Inner Cell Mass. Dev. Cell.

[25] Rayon T., Menchero S., Nieto A., Xenopoulos P., Crespo M., Cockburn K., Canon S., Sasaki H., Hadjantonakis A.K., de la Pompa J.L. (2014). Notch and Hippo Converge on Cdx2 to Specify the Trophectoderm Lineage in the Mouse Blastocyst. Dev. Cell.

[26] Valis K., Novak P. (2020). Targeting ERK-Hippo Interplay in Cancer Therapy. Int. J. Mol. Sci..

[27] Luo X., Zhang R., Lu M., Liu S., Baba H.A., Gerken G., Wedemeyer H., Broering R. (2021). Hippo Pathway Counter-Regulates Innate Immunity in Hepatitis B Virus Infection. Front. Immunol..

[28] Yu F.X., Zhao B., Guan K.L. (2015). Hippo Pathway in Organ Size Control, Tissue Homeostasis, and Cancer. Cell.

[29] Koo J.H., Guan K.L. (2018). Interplay between YAP/TAZ and Metabolism. Cell Metab..

[30] Anani S., Bhat S., Honma-Yamanaka N., Krawchuk D., Yamanaka Y. (2014). Initiation of Hippo Signaling Is Linked to Polarity Rather than to Cell Position in the Pre-Implantation Mouse Embryo. Development.

[31] Meng Z., Moroishi T., Guan K.L. (2016). Mechanisms of Hippo Pathway Regulation. Genes Dev..

[32] Saha B., Ganguly A., Home P., Bhattacharya B., Ray S., Ghosh A., Rumi M.A.K., Marsh C., French V.A., Gunewardena S. (2020). TEAD4 Ensures Postimplantation Development by Promoting Trophoblast Self-Renewal: An Implication in Early Human Pregnancy Loss. Proc. Natl. Acad. Sci. USA.

[33] Meinhardt G., Haider S., Kunihs V., Saleh L., Pollheimer J., Fiala C., Hetey S., Feher Z., Szilagyi A., Than N.G. (2020). Pivotal Role of the Transcriptional Co-Activator YAP in Trophoblast Stemness of the Developing Human Placenta. Proc. Natl. Acad. Sci. USA.

[34] Wang H., Xu P., Luo X., Hu M., Liu Y., Yang Y., Peng W., Bai Y., Chen X., Tan B. (2020). Phosphorylation of Yes-Associated Protein Impairs Trophoblast Invasion and Migration: Implications for the Pathogenesis of Fetal Growth Restrictiondagger. Biol. Reprod..

[35] Liu R., Wei C., Ma Q., Wang W. (2020). Hippo-YAP1 Signaling Pathway and Severe Preeclampsia (SPE) in the Chinese Population. Pregnancy Hypertens..

[36] Sun M., Na Q., Huang L., Song G., Jin F., Li Y., Hou Y., Kang D., Qiao C. (2018). YAP Is Decreased in Preeclampsia and Regulates Invasion and Apoptosis of HTR-8/SVneo. Reprod. Sci..

[37] Gill M.K., Christova T., Zhang Y.Y., Gregorieff A., Zhang L., Narimatsu M., Song S., Xiong S., Couzens A.L., Tong J. (2018). A Feed Forward Loop Enforces YAP/TAZ Signaling during Tumorigenesis. Nat. Commun..

[38] Watanabe Y., Miyasaka K.Y., Kubo A., Kida Y.S., Nakagawa O., Hirate Y., Sasaki H., Ogura T. (2017). Notch and Hippo Signaling Converge on Strawberry Notch 1 (Sbno1) to Synergistically Activate Cdx2 during Specification of the Trophectoderm. Sci. Rep..

[39] Soncin F., Parast M.M. (2020). Role of Hippo Signaling Pathway in Early Placental Development. Proc. Natl. Acad. Sci. USA.

[40] Liu Y., Fan X., Wang R., Lu X., Dang Y.L., Wang H., Lin H.Y., Zhu C., Ge H., Cross J.C. (2018). Single-Cell RNA-Seq Reveals the Diversity of Trophoblast Subtypes and Patterns of Differentiation in the Human Placenta. Cell Res..

[41] Ray S., Saha A., Ghosh A., Roy N., Kumar R.P., Meinhardt G., Mukerjee A., Gunewardena S., Kumar R., Knöfler M. (2022). Hippo Signaling Cofactor, WWTR1, at the Crossroads of Human Trophoblast Progenitor Self-Renewal and Differentiation. Proc. Natl. Acad. Sci. USA.

[42] Cheng J.-C., Fang L., Li Y., Thakur A., Hoodless P.A., Guo Y., Wang Z., Wu Z., Yan Y., Jia Q. (2021). G Protein-Coupled Estrogen Receptor Stimulates Human Trophoblast Cell Invasion via YAP-Mediated ANGPTL4 Expression. Commun. Biol..

[43] Cao Z., Xu T., Tong X., Wang Y., Zhang D., Gao D., Zhang L., Ning W., Qi X., Ma Y. (2019). Maternal Yes-Associated Protein Participates in Porcine Blastocyst Development via Modulation of Trophectoderm Epithelium Barrier Function. Cells.

[44] Totaro A., Panciera T., Piccolo S. (2018). YAP/TAZ Upstream Signals and Downstream Responses. Nat. Cell Biol..

[45] Zhang J., Mo H.-Q., Tian F.-J., Zeng W.-H., Liu X.-R., Ma X.-L., Li X., Qin S., Fan C.-F., Lin Y. (2018). EIF5A1 Promotes Trophoblast Migration and Invasion via ARAF-Mediated Activation of the Integrin/ERK Signaling Pathway. Cell Death Dis..

[46] Piccinni M.P., Raghupathy R., Saito S., Szekeres-Bartho J. (2021). Cytokines, Hormones and Cellular Regulatory Mechanisms Favoring Successful Reproduction. Front. Immunol..

[47] Nair R.R., Verma P., Singh K. (2017). Immune-Endocrine Crosstalk during Pregnancy. Gen. Comp. Endocrinol..

[48] Maliqueo M., Echiburu B., Crisosto N. (2016). Sex Steroids Modulate Uterine-Placental Vasculature: Implications for Obstetrics and Neonatal Outcomes. Front. Physiol..

[49] Zhang Y., Jin S. (2019). Mitigating Placental Injuries through Up-Regulating DAF in Experimental APS Mice: New Mechanism of Progesterone. Clin. Exp. Immunol..

[50] Lan C., Cao N., Chen C., Qu S., Fan C., Luo H., Zeng A., Yu C., Xue Y., Ren H. (2020). Progesterone, via Yes-Associated Protein, Promotes Cardiomyocyte Proliferation and Cardiac Repair. Cell Prolif..

[51] Suzuki D., Okura K., Nagakura S., Ogawa H. (2022). CDX2 Downregulation in Mouse Mural Trophectoderm during Peri-implantation Is Heteronomous, Dependent on the YAP-TEAD Pathway and Controlled by Estrogen-induced Factors. Reprod. Med. Biol..

[52] Schreiber K., Breen K., Cohen H., Jacobsen S., Middeldorp S., Pavord S., Regan L., Roccatello D., Robinson S.E., Sciascia S. (2017). HYdroxychloroquine to Improve Pregnancy Outcome in Women with AnTIphospholipid Antibodies (HYPATIA) Protocol: A Multinational Randomized Controlled Trial of Hydroxychloroquine versus Placebo in Addition to Standard Treatment in Pregnant Women with Antiphospholipid Syndrome or Antibodies. Semin. Thromb. Hemost..

[53] Alvarez A.M., Mulla M.J., Chamley L.W., Cadavid A.P., Abrahams V.M. (2015). Aspirin-Triggered Lipoxin Prevents Antiphospholipid Antibody Effects on Human Trophoblast Migration and Endothelial Cell Interactions. Arthritis Rheumatol..

