# Supplementary material for: Inactivation of Yes-Associated Protein Mediates Trophoblast Dysfunction: A New Mechanism of Pregnancy Loss Associated with Anti-Phospholipid Antibodies?
Source: Biomedicines. 2022 Dec 19;10(12):3296. doi: 10.3390/biomedicines10123296 (PMC9776042; doi:10.3390/biomedicines10123296)
Supplement: Supplementary file 1 [file biomedicines-10-03296-s001.zip › biomedicines-2060940-supplementary.pdf]

Table S1. Clinical and laboratory features of healthy women and aPL women carriers.

Healthy Women	Age	aCL IgG (GPLU/mL)	aβ2GPI IgG (AU/mL)	Clinical Features
1	34	–	–	Gestation 22week
2	31	–	–	Gestation 16week
3	30	–	–	Gestation 11week+1day
4	28	–	–	Non-pregnancy
5	29	–	–	Non-pregnancy
6	26	–	–	Non-pregnancy
aPL Carriers	Age	aCL IgG (GPLU/mL) ⁋	aβ2GPI IgG (AU/mL) ⁋	Clinical Features
1[†]	33	13.9	–	Gestation 23 week, threatened abortion early embryo retardation
2	29	90.4	–	Gestation 17 week, spontaneous pregnancy loss
3	33	53.3	36.4	Gestation 10 week+3 days, Recurrent pregnancy loss
4	32	68	76.4	Spontaneous pregnancy loss, Cornual pregnancy, Infertility
5	30	42.7	98	Spontaneous pregnancy loss, Induced labor (fetal anomaly)
6	26	19.8	–	Recurrent pregnancy loss
7[††]	36	>120	168	Sjogren syndrome, Recurrent pregnancy loss
8	29	29.3	–	Spontaneous pregnancy loss, Infertility

†: Carrier 1 positive for aCL IgG and anti-β2GPI IgM/IgA more than 4 times overall this gestation. ††: Carrier 7 also positive for LAC. ⁋: This table only showed one of several serum test results of aPL although patients were detected consecutively positive for aPL at different times.

aCL, anti-cardiolipin antibodies; aβ2GPI, anti-β2-glycoprotein I antibodies; –: negative; GPLU&AU: The aCL IgG and anti-β2GPI antibodies activity were measured by chemiluminescent immunoassay (CLIA) in our hospital clinical laboratory, in IgG phospholipid units (GPLU) for the CL assay and arbitrary units (AU) for the anti-β2GPI assay (an in-house standard of a patient with positive anti-β2GPI).

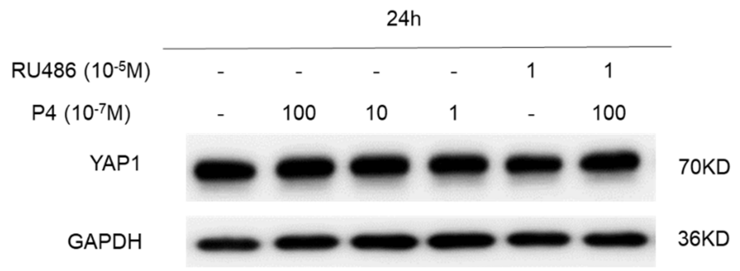

Figure S1. Progesterone treatment for 24 h has no effect on YAP protein expression in vitro. HTR-8 cells were treated with control vehicle (DMSO) or progesterone (P_4) (10^{-7} to 10^{-5}M), alone or in combination with RU486 (10^{-5}M) for 24h. The YAP protein level was analyzed by immunoblotting 24 h after progesterone stimulation. There was no significant change of YAP protein expression. Representative picture of repeated independent experiments was shown.

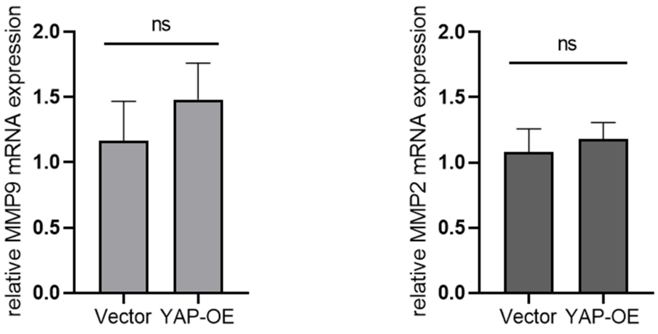

Figure S2. YAP overexpression does not alter the mRNA expression of MMP2 or MMP9. The mRNA levels of MMP2 and MMP9 were quantified by RT-qPCR in cells overexpressed with YAP protein. All values in were presented as the mean± SEM. Student's t test; ns indicates no significance.
